# Long‐term outcomes and complication rates of tooth‐implant‐supported fixed dental prostheses: A retrospective cohort study

**DOI:** 10.1111/jopr.13982

**Published:** 2024-12-10

**Authors:** Simon Dahlgren, Carin Starkhammar Johansson, Shariel Sayardoust

**Affiliations:** ^1^ Department of Prosthodontics Centre for Oral Rehabilitation Linköping Sweden; ^2^ Department of Periodontology Centre for Oral Rehabilitation Linköping Sweden; ^3^ Department of Biomedical and Clinical Sciences Linköping University Linköping Sweden

**Keywords:** complication rate, dental implants, long‐term outcomes, prosthodontics, survival rate, tooth‐implant prostheses

## Abstract

**Purpose:**

To examine the factors influencing the risk of biological and technical complications in tooth‐implant‐supported fixed dental prostheses (T‐I‐FDPs), focusing on location, configuration, and the impact of existing dental health conditions.

**Materials and Methods:**

A retrospective cohort study was conducted, accompanied by a follow‐up clinical and radiological examination, involving 58 patients (37 women, 21 men; mean age: 63.4 years) who had received 68 T‐I‐FDPs at least 5 years earlier, at the Department of Prosthodontics, Centre of Oral Rehabilitation, Region Östergötland, Sweden. Correlations between implant placement specifics, arrangement of teeth and implants, and the presence of root‐filled teeth on the incidence of complications were analyzed.

**Results:**

The analysis highlighted significant complication risk variance, based on the location in the jaw of the implant, with reduced risk for mandibular placements (Hazard ratio [HR] 0.37). Complex arrangements (HR 2.46) and the presence of root‐filled teeth (HR 1.48) were associated with higher complication rates.

**Conclusion:**

This study demonstrates that anatomical considerations and preexisting dental health significantly influence the risk of complications in T‐I‐FDPs. Mandibular implant placements showed a reduced risk of complications compared to maxillary placements. The presence of root‐filled teeth and complex prosthesis configurations were associated with higher complication rates. These findings highlight the need for customized treatment strategies to mitigate risks and enhance long‐term outcomes for patients with T‐I‐FDPs.

Dental implant treatment has been established as a highly predictable method for oral rehabilitation, with long‐term results demonstrating its effectiveness in various scenarios, including total edentulism,^1^ partial edentulism,^2^ and single‐tooth replacement.^3^ The success of these treatments is, however, contingent upon several factors, the most critical being the quality and volume of bone in the implant site area.^4^ Achieving functional and aesthetically pleasing outcomes often necessitates the placement of an adequate number of implants in optimal positions. In cases where such placement is impractical, a combination of teeth and implants to form a tooth and implant‐supported fixed dental prosthesis (T‐I‐FDP) presents a viable alternative.

For long‐span edentulous situations, where placing a sufficient number of supporting implants involves challenges, T‐I‐FDPs have been described as a beneficial treatment alternative. These prostheses not only provide support in long spans but also stabilize teeth with a healthy, albeit reduced, periodontium, reduce the length of bridge spans, and address specific situational prosthetic challenges when a strict tooth‐supported bridge is infeasible.^5^, ^6^

Despite the positive outcomes suggested in the literature with follow‐up periods of up to 10 years, variations in the materials and designs of these prostheses, including the varying use of attachments, have led to complications such as tooth intrusion and screw loosening. These issues are often linked to the movement and stability problems of the attachments.^6^, ^7^, ^8^

Systematic reviews across multiple electronic databases have shown varied outcomes for T‐I‐FDPs, indicating a decline in 10‐year survival rates compared with 5‐year rates, along with an increase in complications.^9^ This underscores the importance of observing caution when choosing T‐I‐FDPs as a treatment option, considering them secondary to other treatments with higher long‐term success rates and fewer complications.

This study aims to conduct a comprehensive retrospective analysis of T‐I‐FDP treatments, evaluating their long‐term durability, efficacy, and associated complications in partially edentulous patients. It contributes to a more detailed understanding of their performance and potential challenges in oral rehabilitation, affirming their role as an essential treatment option in cases where traditional implant strategies might not be applicable.

## MATERIALS AND METHODS

The study design is a retrospective clinical follow‐up study. The Ethical Committee at Linköping University, Linköping, Sweden (reg. no. 2016/152‐31), approved the protocol and the study was conducted in accordance with the 2008 Declaration of Helsinki. This cohort study complied with the Strengthening the Reporting of Observational Studies in Epidemiology (STROBE) criteria, available via the EQUATOR network. All participants received comprehensive information about the study and gave their consent by signing informed consent forms.

**FIGURE 1:**
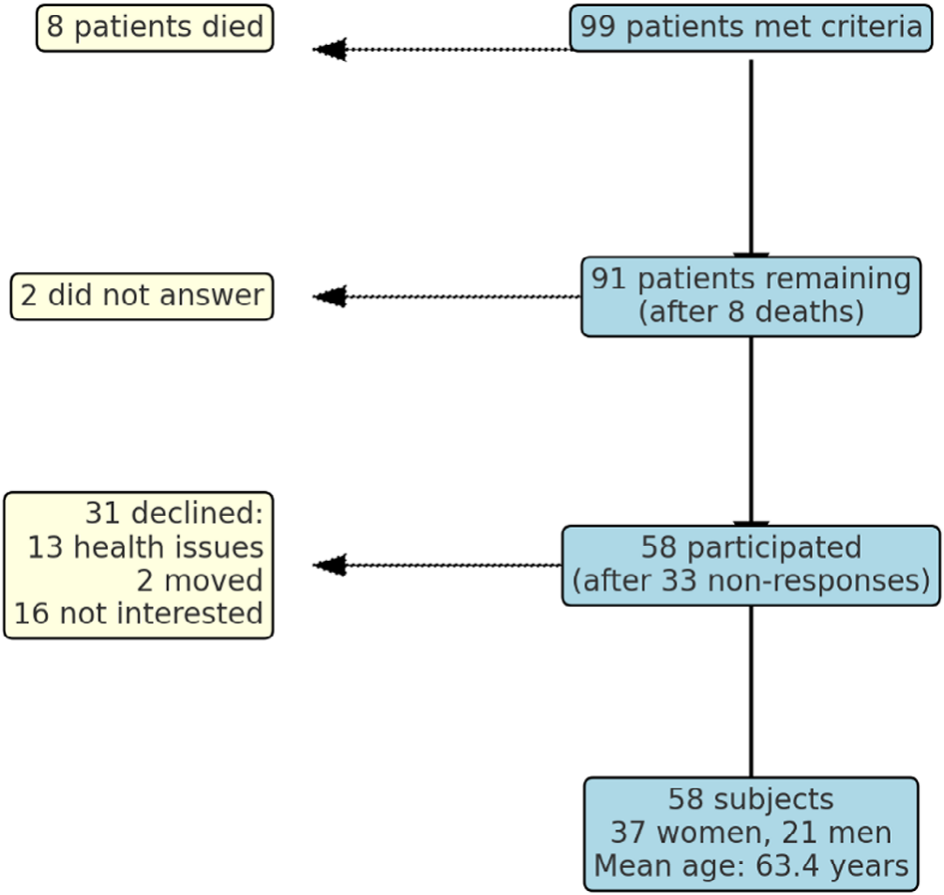
Flowchart inclusion of study subjects.

The inclusion criteria were patients who had received one or several T‐I‐FDPs at the Department of Prosthodontics, Centre of Oral Rehabilitation, Region Östergötland, Sweden at least 5 years earlier. The prostheses were completed between 1998 and 2013. Ninety‐nine patients were identified who met these criteria. Eight patients died; thus, 91 patients were invited to participate, regardless of the number of teeth and implants in the prostheses. Two patients did not respond to the invitation and 31 declined to participate for different reasons: health‐related reasons (*n* = 13); moved to another region (*n* = 2); or not interested (*n* = 16). Finally, the study group consisted of 58 subjects. A flow chart is presented in Figure 1.

Clinical and radiographic data describing the prosthesis design and material, and available follow‐up data during the time of function of the T‐I FPDs were collected from the patient records. Table 1 presents descriptive data for the 68 examined prostheses at baseline.

**TABLE 1:** Descriptive data.

	Mean (number/percentage/year)
Age	63.4
Sex	Female: 62.7% Male: 37.3%
Material	Gold‐ceramic: 98.5%
Position	Mandible: 40.3% Maxilla: 59.7%
Bridge construction	3 units: 33 > 3 units: 35
Implant system	Nobel Biocare: 65 Straumann: 2 AstraTech: 1
Follow‐up time	11.2

*Note*: *n* = 68.

Two calibrated prosthodontics specialists (S.D., M.G.) performed the clinical and radiological examinations. To calibrate the investigators, the first five patients were examined by both specialists, and consensus was reached concerning the clinical and radiological recordings. Data were collected on prosthesis status, technical and biological complications, overall periodontal and cariological status, as well as general health, medication, and smoking habits. The analysis included variables such as patient age, gender, implant placement (mandible vs. maxilla), periodontal diagnosis, number of vital supporting teeth, root‐filled teeth (with and without posts), total number of root‐filled teeth, smoking status, number of implants, marginal bone levels (measured at mesial and distal positions for both mean and maximum values), reason for receiving T‐I‐FDP, distribution of teeth‐implant ratio, implant system used, type of abutment, and opposing dentition. These factors were examined to understand their impact on technical and biological complications over time.

The periodontal examination included the determination of probing pocket depth (PPD), bleeding on probing (BOP%), and plaque index (PLI%). Color‐coded pocket probes were used. The percentage of the total number of sites with plaque or bleeding was recorded for each participant. PPD was measured on four surfaces (mesial, buccal, distal, lingual) on all teeth and implants. The PPD values were recorded if ≥ 4 mm in teeth and for implants to the nearest measured millimeter. Intraoral radiographs on all abutments (teeth and implants) were taken by using parallel and orthoradial projection directions, respectively.

A digital technique with a wire‐connected Schick sensor by Sirona (Germany) was applied. On abutment teeth, alveolar bone loss was measured mesially and distally in millimeters along the root surface, from the most apical part of the restoration to the most coronal level at which the width of the periodontal ligament space was considered normal. On implants, measurements of marginal bone loss mesially and distally were made on radiographs, in millimeters from a reference point related to the implant profile of the actual implant system.

## STATISTICS

Statistical analyses included Kaplan–Meier survival curves and Poisson regression models to study the association between baseline variables and the risk of complications. Continuous variables were presented by means and SDs and categorical variables by frequencies and percentages. Survival curves were also used to describe the material.

**FIGURE 2:**
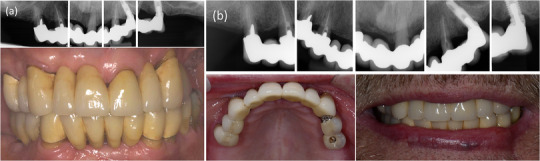
(a) Radiographs and clinical photos of patient‐ID‐105 after 13 years of implant function. (b) Radiographs and clinical photos of the same patient after 20 years of implant function.

The clinical outcomes comprised Technical, Biological, and Technical+Biological complications after surgery. An extension of Poisson regression models^10^, ^11^ was used to study the association between baseline variables and the risk of complications. In contrast to logistic regression, the Poisson regression uses the length of each individual's follow‐up period, and the hazard function is assumed to be exp (*β*_0_ + *β*_1_ · variable of interest). The observation period of each participant was divided into intervals of 1 month. One complication per person and the time to the first complication was counted, and the time at risk was censored at the time of the first complication of the current kind, loss to follow‐up, death, or at the end of follow‐up. First, each baseline variable was investigated univariably. Subsequently, we used multivariate models with stepwise forward selection with variables with a *p*‐value < 0.10 univariably. The associations between baseline variables and outcomes are presented as a hazard ratio (HR), together with 95% confidence intervals (CI) and *p*‐values. Two‐sided *p*‐values were used for all analyses and *p* < 0.05 was considered statistically significant. A case example is presented in Figure 2.

## RESULTS

A total of 58 patients (37 women, 21 men, mean age 63.4 years) participated. Table 1 presents descriptive data for the 68 examined prostheses.

### Prosthesis‐level outcomes for T‐I‐FDPs

In this study, a prosthesis was defined as original if the same number of teeth and implants were maintained, with the initial prosthetic structure still operational and its design unchanged at the time of assessment. The average duration of implant functionality was recorded at 11.2 years, with a range of 5 to 20 years. Of the 68 prostheses evaluated, 58 were found to be still operational and original, resulting in a survival rate of 85.3%. Notably, 25 of the examined prostheses exhibited no complications, yielding a success rate of 36.8%. Conversely, complications were observed in 33 prostheses, with 10 T‐I‐FDPs experiencing failures, translating to a failure rate of 14.7%. The operational lifespan of these failed T‐I‐FDPs ranged between two and 20 years, with a median of 7 years. Table 2 details the reasons for these 10 failures. Additionally, Table 3 provides insights into the technical and biological complications identified in the 58 functioning prostheses.

**TABLE 2:** Failures on patient level.

ID	Type of complication	Year of failure
1	Veneer fracture	4
2	Cement failure	1
3	Cement failure	2
4	Cement failure Loosening of screw components	17 13
5	Caries	9
6	Other technical complication	11
7	Loosening of screw components Cement failure Caries	8 8 9
8	Cement failure Caries	3 3
9	Cement failure Root fracture	6 6
10	Caries	12

**TABLE 3:** Complications on prostheses level.

Type of complication	Number of prostheses	Year of complication (mean)
Loosening of screw component	7	8.3
Fracture of screw	1	11
Cement failure	10	7.2
Veneer fracture	17	11.6
Other technical complication	2	7
Caries	11	8.9
Endodontic problem	4	5.7
Periodontitis	0	0
Peri‐implantitis	7	7.4
Loss of integration	1	12

In terms of maintenance, all patients followed a standardized follow‐up program tailored to their individual risk levels. Patients with good oral health and low risk were scheduled for dental hygienist visits every 6 months. Those at risk for periodontal or peri‐implant issues were seen more frequently, with appointments every 3 to 4 months depending on their risk assessment. Each visit included professional cleaning and personalized oral hygiene instructions to ensure the optimal maintenance of their prostheses and overall oral health.

## Implant‐level findings

The study identified the loss of one implant before examination, which was reported in the failure statistics. The mean marginal bone loss across the remaining 88 implants, measured from a standard reference point, was found to be 1.3 mm (SD ± 1.4) with a range from 0 to 8 mm, categorizing the implants according to their marginal bone loss in millimeters.

## Regression analysis

The findings highlight critical factors influencing the risk of complications in dental implants. Notably, implant placement in the mandible compared with the maxilla significantly reduced the risk of biological complications, with HR of 0.37 in both univariate (*p* = 0.026) and multivariate analyses (*p* = 0.032). The combination of teeth and implants, specifically comparing configurations of three‐unit prostheses versus > three‐unit prostheses, significantly increased the risk of biological complications (HR 2.46, *p* = 0.025) in univariate analyses, though this significance was not maintained in multivariate analyses.

The presence of root‐filled teeth without posts was associated with an increased risk per additional tooth (HR 1.48, *p* = 0.016) in univariate analyses, with no significant impact in multivariate analyses. Similarly, the total number of root fillings indicated an increased risk per unit (HR 1.35, *p* = 0.017) in the univariate analysis, without significance in the multivariate analysis.

A notable finding was the significant increase in the risk of biological complications with each additional implant, with HRs of 2.02 (*p* < 0.001) in univariate analyses and 1.70 (*p* = 0.0062) in multivariate analyses. Additionally, increased marginal bone loss was associated with a higher risk, showing HRs of 1.73 (*p* < 0.001) and 1.81 (*p* < 0.001) in univariate and multivariate analyses, respectively.

These observations underscore the importance of anatomical and biological differences between jaws, the impact of previous dental health on implant success, and the need for meticulous planning and follow‐up to minimize complications. The study suggests smaller dental bridges are better, while larger, more complex bridges involve greater long‐term risk. This reinforces the idea that the cumulative health history of a patient's teeth, including the number of root fillings and the presence of untreated root‐filled teeth, plays a critical role in the success of dental implants.

## Patient‐level data

Similar trends were observed at the patient level. The presence of root‐filled teeth without posts per additional tooth and the total number of root fillings per unit was associated with an increased risk of biological complications, mirroring the construction‐level findings. Additionally, smoking was identified as a significant risk factor for complications, underscoring the importance of considering patient lifestyle factors in dental implant therapy.

**FIGURE 3:**
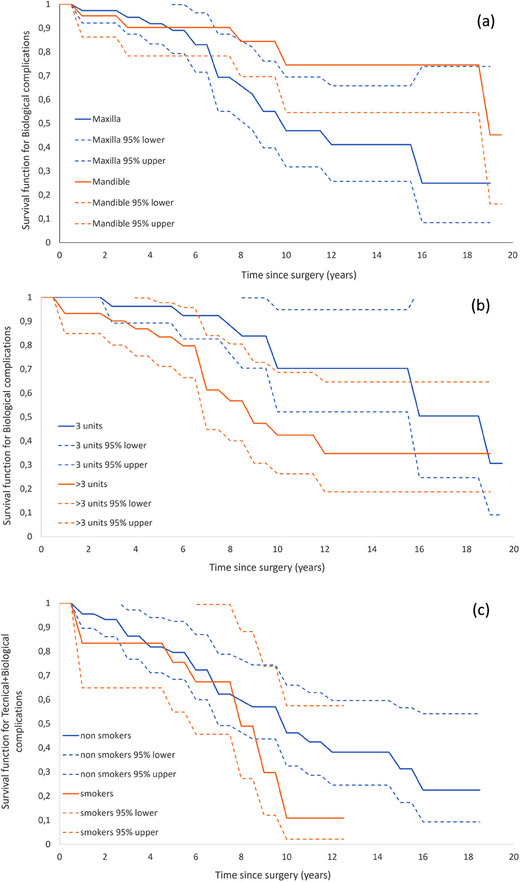
Kaplan–Meier survival curves that compare the survival functions for implants with biological complications over time following surgery under different conditions and with different patient characteristics. Dashed lines are 95% CI. (a) Compares the survival function of biological complications between maxillary and mandibular surgery. Both show a decrease over time, with CI indicated for each. (b) Shows the impact of the number of units involved in the surgery (≤ 3 units vs. > 3 units) on the survival function for biological complications. Again, a larger number of units are associated with poorer survival function, indicating more complications. (c) Illustrates the difference in survival function between smokers and nonsmokers, with nonsmokers demonstrating better survival function, suggesting fewer biological complications. CI, confidence intervals.

These observations underscore the importance of anatomical and biological considerations that are crucial for the success of T‐I‐FDPs, including jaw placement, the complexity of the dental bridge, and the patient's dental history. These findings indicate the need for personalized treatment strategies to optimize long‐term outcomes, emphasizing the significance of both prosthesis and patient‐level data in understanding and mitigating the risks associated with dental implant therapy (Figure 3).

## DISCUSSION

This study presents long‐term outcomes of treatments using T‐I‐FDPs, achieving an 85% survival rate after an average lifespan of 11 years, which is in accordance with findings from Kindberg et al.^12^ and supported by systematic reviews,^9^, ^13^ and positions the findings within the expected performance range of dental prostheses. The comparative analysis with dental‐supported fixed partial dentures and strictly implant‐supported bridges, as outlined in previous studies,^2^, ^14^, ^15^ suggests a comparable reliability and viability of T‐I‐FDPs as a restorative solution.

In our cohort, failure was observed in 10 out of 68 bridges, with a notable pre‐failure functional period averaging 7 years. The feasibility of replacing failed prostheses with identical new ones in three instances highlights the practical reliability and reparability of T‐I‐FDPs, emphasizing their potential as a long‐term treatment option.

This study identified 37% of the bridges as remaining completely free from complications. The most common technical complication, ceramic veneer chip‐off fractures, echoes the complication rates of fixed prosthodontics with porcelain facades documented in the literature.^16^, ^17^, ^18^ Despite their frequency, many of these complications were clinically minor, often going unnoticed by patients.

Biological complications, particularly those affecting the supporting tooth, such as caries, root fractures, and endodontic issues, presented significant threats to the longevity of T‐I‐FDPs. Caries emerged as the primary cause of biological complications in failed cases, corroborating findings from prior research.^19^, ^20^ Moreover, our observation of a single implant loss and the variability in marginal implant bone loss further agree with earlier studies, enhancing our understanding of the biological challenges associated with these prostheses.

An intriguing aspect of this study was the discovery that 80% of the failed bridges had one or more abutments that underwent root canal treatment, highlighting the risk of root fractures—a concern also noted by Nickenig et al.^21^ This observation potentially links implant‐associated vertical root fractures with endodontically treated teeth, indicating a need for further investigation.^22^

The findings provide insight into the multifaceted factors influencing the risk of biological complications in dental implants. Notably, implant placement in the mandible versus the maxilla showed a differential risk, possibly due to anatomical and biological variations affecting healing and integration. This observation is critical for clinical decision‐making and to optimize patient outcomes.

The configuration of teeth and implants was identified as a significant factor in the risk of complications. Complex dental bridges, which may alter masticatory force distribution, were associated with a higher risk of biological complications. This underscores the importance of careful planning and the potential benefits of simpler configurations.

The presence of root‐filled teeth without posts and the total number of root fillings were linked to an increased risk of complications. These findings highlight the impact of dental disease and treatment history on the structural integrity of teeth and surrounding bone, emphasizing the need for a comprehensive oral health assessment in implant planning.

The study by Cordaro et al.^23^ and our study both highlight the importance of considering the periodontal and structural status of abutment teeth in prosthesis design and placement execution. Our findings, alongside those of Cordaro et al., contribute to a nuanced understanding of the mechanical and biological interactions in mixed tooth‐implant‐supported reconstructions, underscoring the necessity of a holistic assessment of supporting structures.

Similarly, this study agrees with Brägger et al.^24^ in stressing the importance of meticulous planning and the consideration of specific risk factors for the success of prostheses. The broader temporal scope extends the discussion on long‐term implications and factors influencing complication risks, complementing the immediate postoperative challenges detailed by Brägger et al.

The long‐term outcome study by Fardal and Linden^25^ and our findings both emphasize careful treatment selection and planning in periodontally compromised patients, highlighting the viability of dental restorations when integrating teeth and implants as abutments.

Both our study and that of Chrcanovic et al.^26^ underline the complexity of using combined tooth‐implant supports in prosthetic dentistry. While Chrcanovic et al. focused on technical complications and failure rates, providing a detailed survival analysis over 2 decades, our study offers insights into anatomical and dental health considerations that influence implant success. The attention to bruxism in the work of Chrcanovic et al. as a significant risk factor adds a crucial dimension to the multifactorial nature of dental implant therapy success, emphasizing the importance of comprehensive patient assessments.

The recent prospective evaluation by Altayyar et al.^27^ provides comparative insights into the functionality of the implants and patient satisfaction across different types of three‐unit posterior FDPs, including tooth‐implant‐supported FDPs. Despite the biomechanical differences inherent in combinations of natural teeth and implants, these configurations achieved functionally acceptable outcomes in terms of maximum biting force and high levels of patient satisfaction. This underscores the critical importance of not only technical performance but also of the patient's perception of functionality and aesthetics for the success of prosthetic solutions.

A pivotal finding from our study is the relationship between the number of implants and the risk of biological complications and an increase in marginal bone loss. This suggests that the risk escalates with the number of implants used, possibly due to increased surgical trauma, alterations in oral biomechanics, or challenges of maintaining oral hygiene around multiple implants. Understanding this relationship is crucial for planning and executing implant treatments, as it underscores the complexity of achieving optimal implant integration and the necessity of meticulous postoperative care and monitoring.

This study presents several limitations inherent to retrospective analyses and case studies. Retrospective studies depend on historical data which can introduce biases due to potential inaccuracies or incompleteness in patient records. The lack of randomization and control groups further limits the ability to establish causality, as observed outcomes might be influenced by uncontrolled confounding factors.

The variability in prosthesis designs and patient conditions presents another significant limitation. Our study population comprised individuals with differing levels of dental health and a variety of prosthetic designs, leading to potential confounding factors that are challenging to control. Such diversity, while reflective of real‐world scenarios, complicates the interpretation of results and their generalizability to a broader population.

Additionally, this study is based on a specific cohort treated at a single center, which may not represent the broader population. The findings, while insightful, come from a case study approach rather than a randomized controlled trial (RCT), limiting the strength of the evidence. Controlled clinical trials are essential to confirm the generalizability and reliability of these findings, as they provide a more rigorous framework for assessing treatment outcomes.

Despite these limitations, this study offers valuable insights into the factors influencing the long‐term success and complications of tooth‐implant‐supported fixed dental prostheses (T‐I‐FDPs). It underscores the importance of meticulous planning, anatomical considerations, and individualized treatment strategies. Future research should aim to conduct larger, RCTs with standardized protocols to provide more definitive evidence. Investigating the impact of newer materials and technologies on the long‐term performance and patient satisfaction of T‐I‐FDPs will also be crucial in advancing the field of prosthodontics.

## CONCLUSIONS

This comprehensive analysis emphasizes the importance of anatomical considerations, previous dental health, and meticulous planning for the success of T‐I‐FDPs. The study demonstrates that anatomical considerations and preexisting dental health significantly influence the risk of complications in T‐I‐FDPs. Mandibular implant placements showed a reduced risk of complications compared to maxillary placements. The presence of root‐filled teeth and complex prosthesis configurations were associated with higher complication rates. The study advocates personalized treatment strategies to mitigate complications and improve long‐term outcomes. The adaptability of tooth‐implant‐supported FDPs emerges as a valuable alternative in complex cases, demonstrating the potential of these treatments to address the unique needs of patients with complicated dental conditions.

Considering these findings, future research should continue to explore the specific challenges and advantages of tooth‐implant‐supported FDPs. Investigating long‐term clinical performance, patient satisfaction, and the integration of new materials and technologies will be crucial to enhance our understanding and improve clinical outcomes for patients. Moreover, the development of guidelines for optimal planning and execution of these prostheses could significantly contribute to reducing complications and increasing the longevity of dental restorations.

This study contributes significantly to the existing body of knowledge, offering insights that can aid in the refinement of clinical practices and the formulation of strategies aimed at optimizing the success of tooth‐implant‐supported FDPs. As the dental field evolves, embracing a patient‐centered approach that incorporates the latest research findings will be paramount to achieving the best possible outcomes for patients seeking restorative dental treatments. In conclusion, our analysis underlines the importance of anatomical considerations, prior dental health, and careful planning for the success of dental implants. The study advocates customized treatment strategies to reduce complications and enhance long‐term outcomes. Notably, this treatment approach emerges as a valuable alternative in complex cases where conventional prostheses are not feasible, offering a viable solution for challenging dental reconstructions. This adaptability underscores the treatment's utility in addressing the unique needs of patients with complicated dental conditions.

## CONFLICT OF INTEREST STATEMENT

The authors declare no conflicts of interest.
